# Dated Phylogenies of the Sister Genera *Macaranga* and *Mallotus* (Euphorbiaceae): Congruence in Historical Biogeographic Patterns?

**DOI:** 10.1371/journal.pone.0085713

**Published:** 2014-01-17

**Authors:** Peter C. van Welzen, Joeri S. Strijk, Johanna H. A. van Konijnenburg-van Cittert, Monica Nucete, Vincent S. F. T. Merckx

**Affiliations:** 1 Naturalis Biodiversity Center, sector Herbarium, Leiden, The Netherlands; 2 Institute Biology Leiden, Leiden University, Leiden, The Netherlands; 3 Key Laboratory of Tropical Forest Ecology, Xishuangbanna Tropical Botanical Garden, Chinese Academy of Sciences, Menglun, Mengla, Yunnan Province, P.R. China; Onderstepoort Veterinary Institute, South Africa

## Abstract

Molecular phylogenies and estimates of divergence times within the sister genera *Macaranga* and *Mallotus* were estimated using Bayesian relaxed clock analyses of two generic data sets, one per genus. Both data sets were based on different molecular markers and largely different samples. Per genus three calibration points were utilised. The basal calibration point (crown node of all taxa used) was taken from literature and used for both taxa. The other three calibrations were based on fossils of which two were used per genus. We compared patterns of dispersal and diversification in *Macaranga* and *Mallotus* using ancestral area reconstruction in RASP (S-DIVA option) and contrasted our results with biogeographical and geological records to assess accuracy of inferred age estimates. A check of the fossil calibration point showed that the Japanese fossil, used for dating the divergence of *Mallotus*, probably had to be attached to a lower node, the stem node of all pioneer species, but even then the divergence time was still younger than the estimated age of the fossil. The African (only used in the *Macaranga* data set) and New Zealand fossils (used for both genera) seemed reliably placed. Our results are in line with existing geological data and the presence of stepping stones that provided dispersal pathways from Borneo to New Guinea-Australia, from Borneo to mainland Asia and additionally at least once to Africa and Madagascar via land and back to India via Indian Ocean island chains. The two genera show congruence in dispersal patterns, which corroborate divergence time estimates, although the overall mode and tempo of dispersal and diversification differ significantly as shown by distribution patterns of extant species.

## Introduction

**Figure 1:**
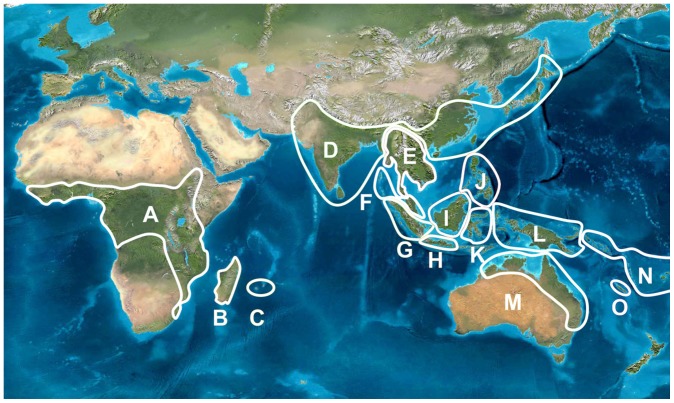
Subdivision of the combined distributions of *Macaranga* and *Mallotus* based on the presence of endemic species: A  =  Tropical Africa; B  =  Madagascar; C  =  Mascarene Islands; D  =  Pakistan-India (not Andaman/Nicobar Isl.) to S. China and Japan; E  =  Thailand (not Peninsular part), Laos, Cambodia, Vietnam; F  =  Peninsular Thailand, Malay Peninsula, Andaman and Nicobar Islands; G  =  Sumatra; H  =  Java; I  =  Borneo; J  =  Philippines; K  =  Sulawesi; L  =  Moluccas, New Guinea; M  =  Australia; N  =  West Pacific island chains; O  =  New Caledonia.

**Figure 2:**
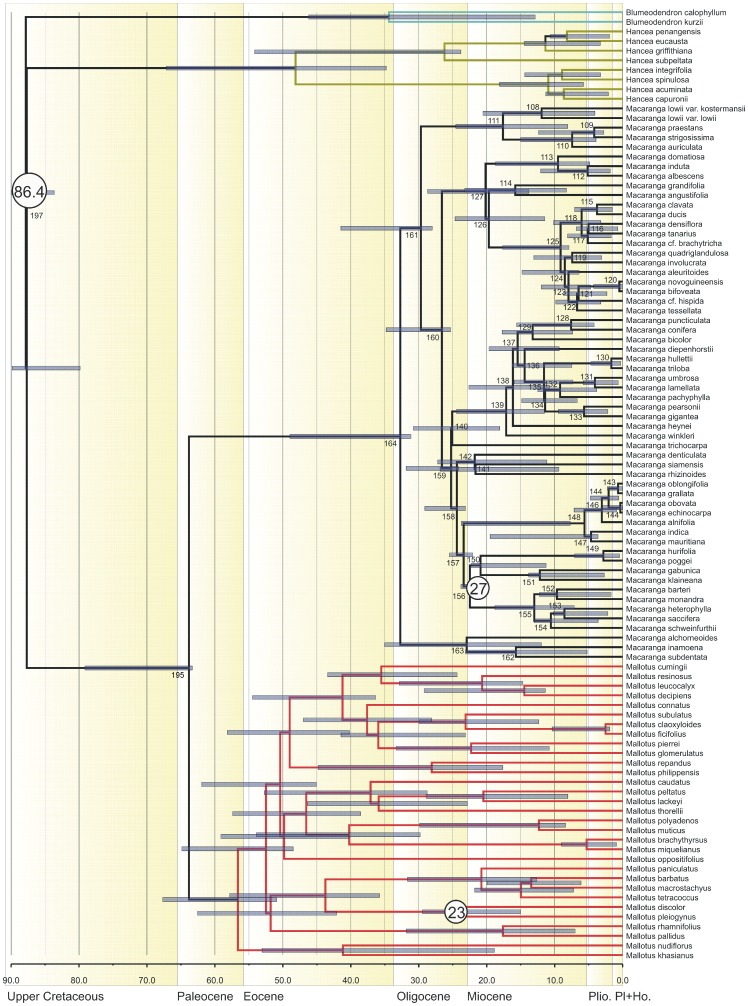
Chronogram resulting from analysis of data set 1 (mainly *Macaranga* and a small sample of *Mallotus*) using BEAST. The three calibration points are indicated with their estimated mean age (circles with numbers). Node bars show the 95% Height of the Posterior Density interval. *Hancea* and *Blumeodendron* were used as outgroups.

**Figure 3:**
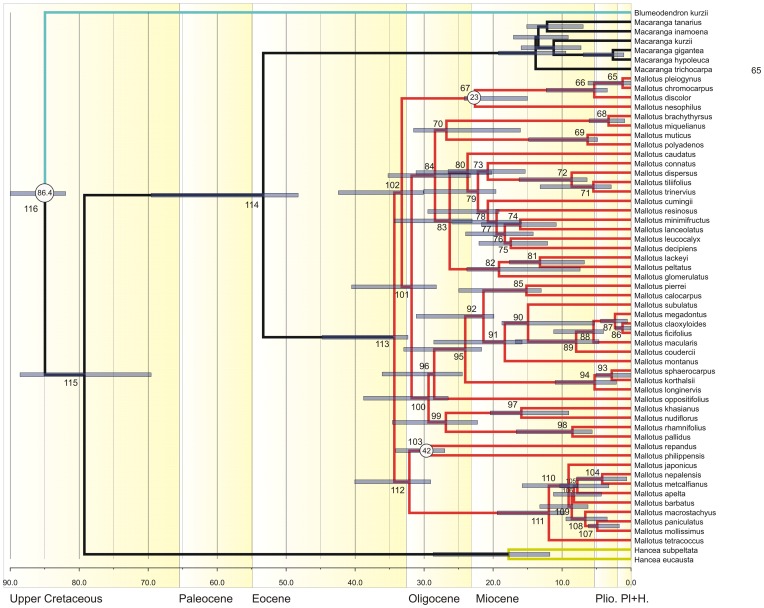
Chronogram resulting from analysis of data set 2 (a large sample of *Mallotus*) using BEAST. The three calibration points are indicated with their estimated mean age (circles with numbers). Node bars show the 95% Height of the Posterior Density interval. *Hancea* and *Blumeodendron* were used as outgroups.

*Macaranga* Thouars and *Mallotus* Lour. are monophyletic sister genera in the Euphorbiaceae or Spurge family [1], [2] comprising 240–282 and 110–135 species respectively [3], [4]. Most species are shrubs to small trees and the genera show a remarkable resemblance in their phylogeny, habit, ecological shifts and geographical distribution. Most species are found in the Malay Archipelago (Malesia) [5], but the genera range from Africa to southeast Asia to Australia and the west Pacific (Fig. 1). Morphologically the only consistent difference between the genera is the number of thecae in the anthers (3 or 4 in *Macaranga*, 2 in *Mallotus*). Other differences include presence of stellate hairs in *Mallotus* and their general absence in *Macaranga*, opposite leaves in many *Mallotus* species, and generally raceme-like inflorescences and more stamens per staminate flower in *Mallotus* and more panicle-like inflorescences and fewer stamens in *Macaranga*. The species that are part of the first diverging lineages of each clade [1] are mainly found in primary rain forest and typically have relatively narrow leaves (e.g., the group of *Macaranga lowii* King ex Hook.f. to *M. strigosissima* Airy Shaw in Fig. 2, the clade of *Mallotus pleiogynus* Pax & K.Hoffm. up to *M. nesophilus* Müll.Arg. in Fig. 3). Later diverging lineages in both clades contain pioneer species with a preference for secondary environments, with larger leaf surface and increased lamina width (e.g., *Macaranga tanarius* (L.) Müll.Arg., *Mallotus barbatus* Müll.Arg.). As such, a number of species in both genera are good indicators for either undisturbed, primary rain forest or various kinds of disturbance (selective logging, burning, repetitive burning) [6]. The geographic distribution of both genera is roughly identical, ranging from Central Africa and Madagascar to India and Southeast Asia, then throughout Malesia [5] to Australia and the West Pacific. *Mallotus* reaches higher latitudes in Asia (up to northern India and Japan) than *Macaranga*, but the latter is generally more species rich in most shared areas.

A previous study inferred the ancestral area of both genera in Asia with one or two dispersal events in both genera from Asia to Africa [1]. The presence of palaeotropical intercontinental disjunctions (PIDs) is interesting, because four competing theories exist to explain them: (1) the “out of India” hypothesis, whereby a rafting Indian plate transported taxa from what is presently Africa to Asia [7], [8]; (2) dispersal via boreotropical forests of the Palaeocene and Eocene [9]–[11]; (3) long-distance dispersal over the Indian Ocean [12], [13], for instance via the various island arcs [1], [14]; and (4) migration overland between Africa and Asia across Arabia and Southwest Asia during a warm phase in the early to middle Miocene [15]. This study will contribute to this discussion.

The two genera, perhaps due to their shared evolutionary background, seemingly diversified and responded in similar ways to temporal changes in ecology and geology through time (concordant evolution). We tested this hypothesis by estimating divergence times for both genera and by reconstructing their historical biogeography. For this purpose we used two already constructed data sets, data set 1 with *Macaranga* and *Mallotus* data [1] and data set 2 with predominantly *Mallotus* data [2]). Both sets have different molecular markers and are thus independent to a high degree. Subsequently, lineages-through-time (LTT) plots were used to compare the timing and tempo of diversification in each genus and historical biogeographical analyses were undertaken to test ancestral area reconstructions and their timing against data from the geological records. In the light of the results, various scenarios for long distance dispersal to Africa and E. Malesia and Australia are discussed.

## Materials and Methods

### Sampling

The aligned *Macaranga* DNA sequence data (data set 1) were obtained from [1], 57 species (ca. 20% of all species), and *Mallotus* (data set 2) from [2], 50 species (ca. 37% of all species). Appendix S1 contains details of the taxa sampled, additional accession and voucher information can be found in [1] for *Macaranga* and in [2] for *Mallotus*. Two clades of recently speciated Bornean *Macaranga* species, all obligate myrmecophytic species, were not included in data set 1; information about their phylogenetic relationships can be found in [16], [17]. Both data sets contain representatives of the other genus. Species of *Blumeodendron* Kurz and *Hancea* Seem. were used as outgroups for both data sets. The aligned sequences are available via the first author and for data set 1 also via the journal website as additional material at www.amjbot.org/content/94/10/1726/suppl/DC1. The nomenclature of some taxa has since been updated to the presently accepted names: the genera *Neotrewia* Pax & K.Hoffm., *Octospermum* Airy Shaw and *Trewia* L. are included in *Mallotus* [18]; *Cordemoya* Baill., *Deuteromallotus* Pax & K.Hoffm. and the species *Mallotus eucaustus* Airy Shaw, *M. griffithianus* (Müll.Arg.) Hook.f., *M. penangensis* Müll.Arg., and *M. subpeltatus* (Blume) Müll.Arg. are included in *Hancea* [19]; and *Macaranga repandodentata* Airy Shaw is synonymized with *Macaranga strigosissima* [20]. Model partitioning for data set 1 followed [1]: ITS (727 bases): GTR+G+I, *phyC* (644 bases): HKY+G, *trnL-F* (1164 bases): GTR+G, *ncpGS* (962 bases): HKY+G; and for data set 2 followed [2]: *matK* (1983 bases): GTR+G, *gpd* (624 bases): HKY+G.

### Calibration Points

Divergence time estimates were performed with four calibration points, one a secondary calibration (a, below) and three based on fossils (b, c, and d below):

- The crown node of all included taxa (which form a monophyletic group) [21] was selected and assigned a mean age (μ) of 86.4 Ma with a lower and upper bound of 90 and 81 Ma. This is the age of the crown node of the *Acalypa-Suregada* clade in Fig. S30 of the additional material of [22]. Unfortunately, *Macaranga* and *Mallotus* were not sampled in this analysis [22], therefore, as lower bound, the divergence time of all Euphorbiaceae s.s. was taken from the same chronogram and as upper bound the divergence time of the *Acalypha-Moultonianthus* clade. *Macaranga* and *Mallotus* are part of the first two clades and probably also of the last one (compare [22] with [21]).
- Fossil leaves, flowers, fruits and pollen from the Oligocene/Miocene (μ = 23 Ma, between 31–15 Ma [23]) of southern New Zealand were reported by [23] and linked to *Mallotus nesophilus* by [24] based on leaf anatomical, inflorescence and fruit features. In data set 2 this calibration point is associated with the crown node of the clade *Mallotus chromocarpus* Airy Shaw, *M. discolor* F.Muell. ex Benth., *M. nesophilus* and *M. pleiogynus* Pax & K.Hoffm. (*Octospermum pleiogynum* (Pax & K.Hoffm.) Airy Shaw in [1]. *Mallotus nesophilus* was not sampled in data set 1, but based on [2] it was linked to the crown node of *M. discolor* and *M. pleiogynus*.
- An African fossil described by [25] and considered to most closely resemble *Macaranga kilimandscharica* Pax by [24], μ = 27 Ma (Oligocene; between 32–22 Ma [25]). Again, this species was not included in data set 1, but *M. kilimandscharica* is most likely part of the African clade of *Macaranga barteri* Müll.Arg., *M. gabunica* Prain, *M. heterophylla* (Müll.Arg.) Müll.Arg., *M. hurifolia* Beille, *M. klaineana* Pierre, *M. monandra* Müll.Arg., *M. poggei* Pax, *M. saccifera* Pax, and *M. schweinfurthii* Pax and was attached at the crown node of this clade.
- *Mallotus hokkaidoensis* Tanai is described from the Middle Eocene (48.6–27.3 Ma [26], [27]) from Japan [26], [27]. This species resembles a group of the polyphyletic *Mallotus* ‘section’ *Philippinensis* clades, namely *M. philippensis* (Lam.) Müll.Arg. and *Mallotus repandus* (Rottler) Müll.Arg. [24]. It was used as a calibration point in the analysis of data set 2; μ = 42, between 49–27 Ma).

Each dataset was analysed using three calibration points: a to c were used in the *Macaranga* analysis (data set 1) and a, b and d were used in the *Mallotus* analysis (Set 2). Throughout this paper, we use the geological timescale on the International Stratigraphic Chart by the International Commission on Stratigraphy (based on [28], [29]).

### Analyses

The molecular dating analyses were performed in a Bayesian framework using BEAST 1.7.5 [30]–[32] with input files created using BEAUTi 1.7.5. Taxon names were imported from a nexus format file, one for each set. For data set 1 (*Macaranga*) six monophyletic groups were defined (all taxa with calibration point a, all taxa minus *Hancea*, *Macaranga+Mallotus*, and two groups for the fossil calibrations points b and c); for all, fossil set b and fossil set c the mean ages were given (see above). For data set 2 (*Mallotus*) only three monophyletic groups were defined (groups for calibration points a, b, and d). A random starting tree was selected together with a relaxed, uncorrelated lognormal clock and speciation according to a Yule process [33]. As no indication existed for a distribution type of the fossil ages, the calibration priors were coded as uniform distributions [34] within the time ranges of the fossils (see above), which means that the fossils will act as minimum ages of the clades. All other priors were set to default except ucld.mean, which was also set to uniform. Each analysis employed three MCMCs, run for 40,000,000 generations for data set 1 and 50,000,000 generations for data set 2, whereby every 1,000^th^ tree was saved. Tracer v. 1.5 [35] was used to monitor for adequate mixing of the chains and convergence of the runs. Based on the Tracer output a burn in of 10% was used. Finally, consensus trees with mean age estimates were calculated with TreeAnnotator 1.7.5 (BEAST package) and visualised with Figtree 1.4.0 [36]. For each data set all MCMC runs produced the same MCC tree, thus only the last run in each data set was used for the historical biogeographical analyses.

We visually assessed the temporal accumulation of lineages in *Macaranga* and *Mallotus* by plotting lineages-through-time (LTT) based on the excised ingroups from our BEAST MCMC chronogram in GENIE v3.0 [37]. To evaluate the effects of incomplete taxon sampling on the slope of our empirical LTT curves, we generated 1000 simulated trees based on the extant number of recognized species in each genus (*Macaranga*: 240, *Mallotus*: 110) using a constant rates birth-death model in PHYLOGEN v1.1 [38]. A number of terminals equal to the number of species in each genus not sampled in our data sets was selected randomly and pruned from each tree, and branch lengths were rescaled to the crown age of the clades using TREE-EDIT v1.0 [38]. Simulated trees were used to construct mean LTT curves and 95% confidence intervals for comparison with the empirical curves derived for *Macaranga* and *Mallotus*.

The S-DIVA (Statistical Dispersal-Vicariance Analysis, modified from DIVA [39]) in the package RASP (Reconstruct Ancestral State in Phylogenies; [40]–[42]) was used to reconstruct the ancestral geographical distributions. The BEAST output files were used as input (trees files and the MCC tree files). The combined distribution of *Macaranga* and *Mallotus* was divided into 15 geographic areas (the maximum number allowed in S-DIVA) based on the presence of several endemic species per area (Fig. 1) and the general use of the Malesian islands as phytogeographic units [43]. The areas used and the distributions of the sampled species are given in Appendix S1. The analysis uses distributions of contemporary species, which does not mean that we automatically assume that continental configurations were similar through time (contra [44]). RASP analysis was conducted with 2, 3, and 4 areas per ancestral node and for data set 1 only the last 10,000 trees of the BEAST analysis were used. Higher numbers of areas per ancestral node resulted in (more) geologically unlikely combinations of areas and considerable increases in computation time.

## Results

### Phylogenetic and molecular dating analyses

Analyses in Tracer showed the effective sampling sizes (ESS) of all parameters exceeded 200, indicating that they are a good representation of the posterior distributions (posterior ESS for data set 1 = 1348, and for data set 2 = 2286). The resulting chronograms are shown in Fig. 2 (data set 1, *Macaranga*) and Fig. 3 (data set 2, *Mallotus*). In both chronograms *Macaranga* and *Mallotus* are sister taxa, and their shared node is dated at 63.82 Ma [79.13–63.33 Ma 95% highest posterior density interval (HPD)] based on data set 1 (195, Fig. 2, Table 1) and somewhat younger based on data set 2 (node 114, Fig. 3, Table 2): 53.32 (HPD 69.57–48.25). The crown node forms the stem nodes for the genus clades. The mean crown node age is 58.5 (HPD 79.13–48.25) Ma. The stem node of both genera together has a mean for both sets of 83.47 (HPD 89.84–69.56) Ma.

**Table 1:** Nodes in the *Macaranga* phylogeny with their estimated mean ages, their variation (95% highest posterior density interval, HPD) and S-DIVA area optimisations with marginal probabilities (MP), in bold selected ones when various area combinations had the same MP.

Node	Mean age	95% HPD	Posterior	S-DIVA area+MP
108	11.91	20.56–4.07	0.55	I = 100
109	4.18	12.40–2.75	0.70	I = 100
110	7.43	15.03–3.91	1.00	I = 100
111	17.61	24.54–8.08	1.00	I = 89.10
112	5.13	12.07–1.81	0.89	L = 100
113	9.51	18.68–4.84	1.00	L = 100
114	15.81	23.23–8.24	0.36	**JKL** = KL = JK = 33.33
115	3.77	7.10–1.51	1.00	L = 100
116	5.01	6.83–0.70	0.36	L = 100
117	5.12	8.13–1.67	0.47	L-100
118	6.03	17.66–7.89	1.00	L-100
119	7.42	13.05–1.67	0.63	L = 100
120	0.48	4.27–0.00	1.00	L = 100
121	6.48	8.72–2.31	0.23	L = 100
122	6.70	9.82–3.21	0.73	L = 100
123	7.96	11.96–4.70	0.94	L = 100
124	8.51	14.81–6.40	0.77	L = 100
125	9.12	17.66–7.89	1.00	L = 100
26	19.68	24.66–11.45	0.76	L = 69.44
127	20.17	28.69–13.78	1.00	L = 94.51
128	7.58	15.60–4.19	0.60	G = 59.69
129	13.23	17.73–7.36	0.24	IJ = 37.30
130	1.66	4.67–0.27	1.00	G = 23.15
131	4.08	5.78–0.64	1.00	I = 100
132	9.19	12.48–3.82	0.95	I = 43.74
133	5.66	9.46–2.17	1.00	I = 51.30
134	11.41	14.84–6.66	0.23	I = 99.74
135	11.57	15.98–7.31	0.52	GI = 61.64
136	14.42	16.29–7.46	0.54	G = 32.77
137	15.44	19.66–9.34	0.93	G = 34.42
138	16.14	22.63–10.80	0.32	G = 23.07
139	17.13	24.47–11.49	1.00	GI = 42.39
140	25.09	30.81–18.08	0.60	G = 25.72
141	21.65	26.85–9.37	0.28	**EGH** = EG = 34.16
142	21.76	27.20–11.14	0.53	E = 21.86
143	0.67	2.22–0.01	0.99	B = 100
144	0.33	2.96–0.03	0.39	B = 100
145	2.06	4.73–0.57	0.31	B = 100
146	3.06	7.14–0.92	1.00	B = 100
147	4.64	19.49–3.61	0.98	**BG** = 2.88, many combinations
148	15.61	23.75–7.68	0.92	B = 5.45
149	2.83	7.06–0.47	1.00	A = 100
150	12.18	13.82–2.70	1.00	A = 100
151	20.90	22.30–11.26	0.75	A = 100
152	9.66	12.27–1.69	1.00	A = 100
153	8.56	10.16–2.16	0.93	A = 100
154	10.55	13.02–3.60	0.98	A = 100
155	12.99	18.78–7.10	1.00	A = 100
156	22.48	23.80–22.00	1.00	A = 100
157	23.39	25.46–22.05	1.00	**ABG** = 3.58; many combinations
158	24.39	29.11–23.11	0.95	G = 12.55
159	25.26	31.86–24.13	0.99	G = 12.51
160	26.60	34.79–25.28	1.00	GL = 10.54; many combinations
161	29.68	41.46–28.01	0.99	I = 13.81
162	15.71	23.12–5.22	1.00	M = 100
163	22.93	35.02–11.92	1.00	MO = 100
164	32.72	48.96–31.14	1.00	**IMO** = 4.89; many combinations
195	63.82	79.13–63.33	1.00	I = 38.42

**Table 2:** Nodes in the *Mallotus* phylogeny with their estimated mean ages, their variation (95% highest posterior density interval, HPD) and S-DIVA area optimisations with marginal probabilities (MP), in bold selected ones when various area combinations had the same MP.

Node	Mean age	95% HPD	Posterior	S-DIVA area+MP
65	1.22	6.24–0.00	0.58	**LM = **L = 50.00
66	5.33	12.29–3.45	0.85	M = 56.40
67	22.62	24.17–15.00	1.00	M = 93.96
68	3.26	6.09–0.92	1.00	I = 99.99
69	6.29	14.84–4.86	0.94	**FIM = 9.03**; many combinations
70	26.76	31.53–16.04	0.81	I = 100.00
71	5.46	13.18–2.88	0.47	L = 2.72
72	8.61	16.23–6.37	0.83	LM = 2.34
73	20.77	26.52–15.34	0.59	**IM** = IJM = IJL = I = IL = 15.26
74	16.06	21.70–10.85	0.76	**EFIJ = 5.34**; many combinations
75	17.43	22.04–12.10	0.72	E = 53.79
76	18.30	23.98–14.19	0.74	E = 17.76
77	19.49	25.98–15.91	0.82	E = 8.03
78	20.76	29.46–19.14	0.17	I = 24.40
79	22.20	30.06–19.58	0.46	I = 73.06
80	23.70	31.16–20.22	0.47	I = 94.17
81	13.21	17.63–6.77	0.89	I = 99.82
82	19.14	23.80–17.39	0.07	EI = 87.08
83	26.29	34.3–23.04	0.12	I = 99.81
84	28.42	35.23–23.31	0.36	I = 92.52
85	15.17	24.96–13.02	0.92	E = 100.00
86	1.24	2.3–0.02	0.67	M = 100.00
87	2.32	4.42–0.54	0.93	M = 93.75
88	5.43	11.21–3.99	0.43	LM = 100.00
89	7.96	16.77–4.65	0.27	ELM = 39.94
90	14.93	18.71–5.29	0.79	ELM = 10.92
91	18.30	28.59–15.80	0.67	**EFL** = EFLM = EFM = 9.20
92	21.39	31.14–19.88	0.70	E = 84.04
93	2.32	5.08–0.00	0.18	G = 17.55
94	5.43	11.01–2.12	0.32	I = 53.98
95	24.03	32.95–21.67	0.26	EI = 41.59
96	28.54	36.08–24.47	0.26	**BE** = AE = ABE = 8.91
97	15.90	20.41–9.03	0.97	E = 69.86
98	8.49	16.63–5.64	1.00	DE = 100.00
99	26.84	34.61–22.26	0.46	E = 56.25
100	29.36	38.78–26.52	0.20	E = 55.68
101	31.81	40.51–28.22	0.37	EI = 48.84
102	33.24	42.43–30.11	0.34	EIM = 26.52
103	29.66	34.13–27.00	1.00	D = 57.52
104	4.18	7.87–0.62	0.36	D = 99.81
105	7.78	10.35–3.22	0.13	D = 99.93
106	8.28	11.24–4.31	0.18	D = 100.00
107	4.90	6.13–1.71	1.00	I = 44.40
108	6.63	9.44–3.46	0.99	I = 44.76
109	8.59	13.22–6.34	0.39	DG = 53.47
110	9.05	15.77–7.74	0.76	D = 80.87
111	11.91	19.38–9.66	1.00	D = 65.36
112	32.13	40.04–29.04	0.97	D = 43.96
113	34.31	44.79–32.35	0.53	I = 13.62
114	53.32	69.57–48.25	1.00	I = 49.39

The crown node age for *Macaranga* (node 164 in Fig. 2, Table 1) is 32.72 (HPD 48.96–31.14) Ma, and for *Mallotus* (node 113 in Fig. 3, Table 2) 34.31 (HPD 44.79–32.35) Ma, similar estimates for both genera in spite of different samples of species and markers.

### Lineages through time plots

**Figure 4:**
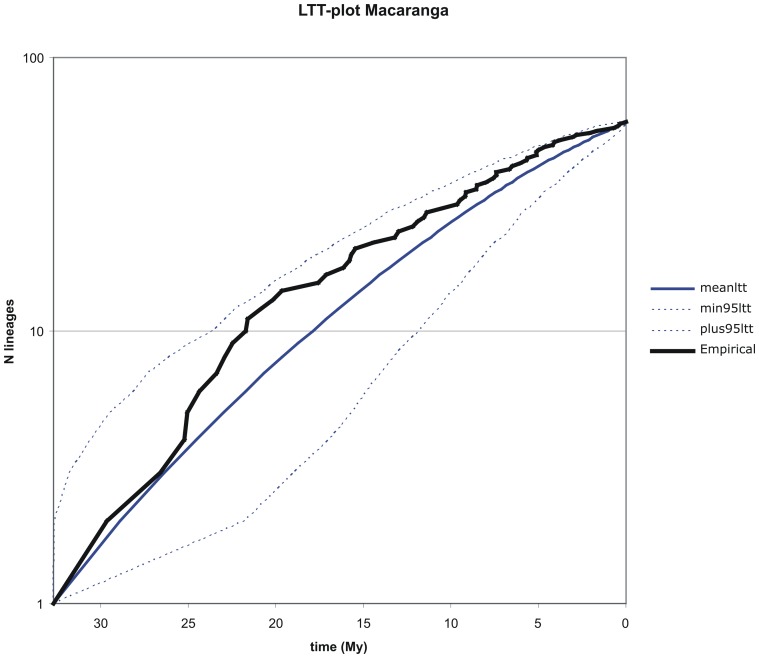
Plot of Lineages Through Time (LTT) for *Macaranga*. Empirical curves (black line) and simulated curves (unbroken blue line) are shown with 95% confidence intervals (dashed blue lines) for the sampled ingroup clade. The constant rate model is rejected when the empirical curve falls outside the 95% confidence interval.

**Figure 5:**
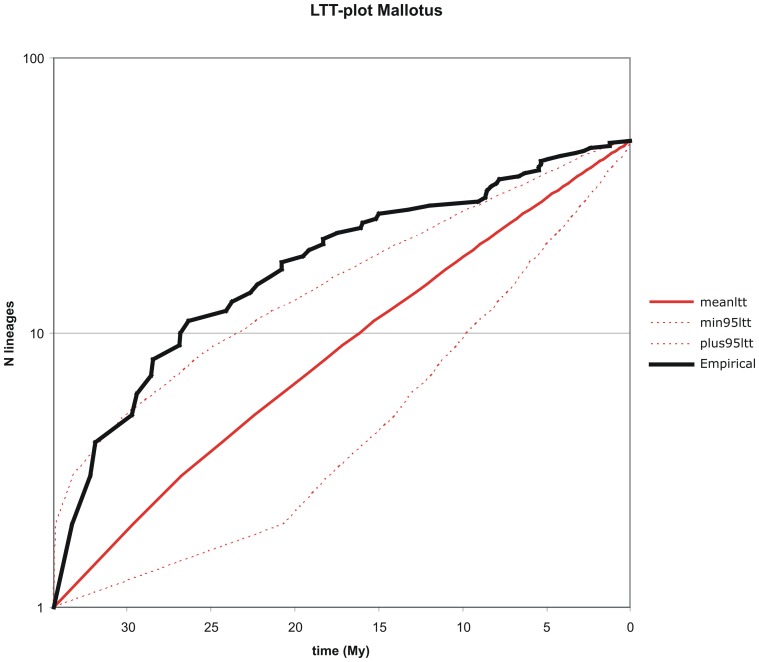
Plot of Lineages Through Time (LTT) for *Mallotus*. The empirical (black line) and simulated curves (unbroken red line) are shown with 95% confidence intervals (dashed red lines) for the sampled ingroup clade. The constant rate model is rejected when the empirical curve falls outside the 95% confidence interval.

The LTT curve for *Macaranga* (Fig. 4) shows considerable variation over time and, except for one instance, a small peak around 20 Ma, roughly conforms to a constant diversification rate model as delimited by the simulated 95% confidence interval. The empirical curve describing the changes in diversification rate over time in *Mallotus* (Fig. 5) is almost entirely located outside the 95% confidence interval pertaining to a constant diversification rate model, indicating that for this genus this model is rejected. Several sharp changes in diversification rate can be seen over time. From the onset of diversification in the Early Eocene the curve shows a gradual decline towards the present. The difference between the genera can also be seen in Fig. 2. Diversification in *Mallotus* starts earlier than in *Macaranga*, but also decreases earlier.

### Historical Biogeography

**Figure 6:**
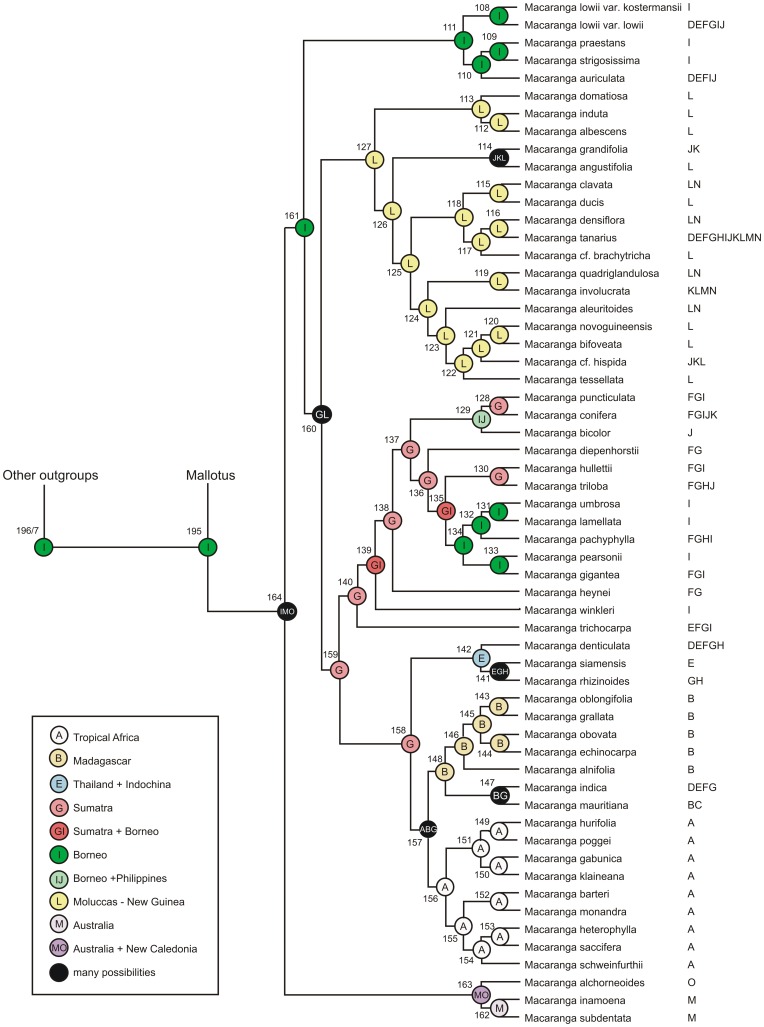
RASP analysis showing the most likely area optimizations for nodes on the molecular phylogeny for *Macaranga* (data set 1). Area nomenclature follows Fig. 1.

**Figure 7:**
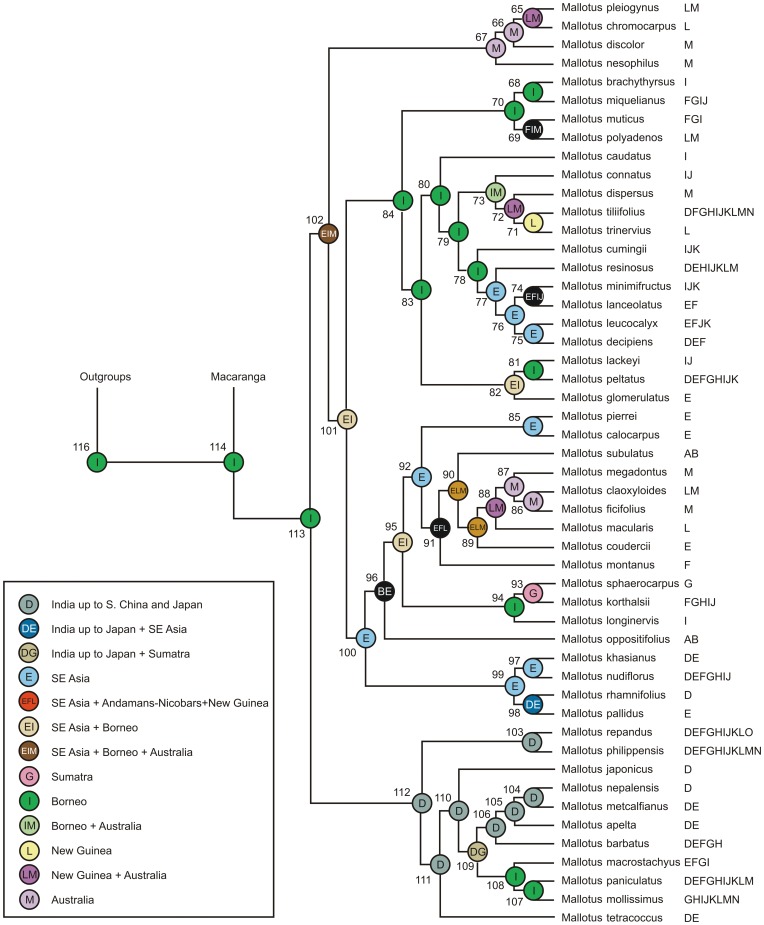
RASP analysis showing the most likely area optimizations for nodes on the molecular phylogeny for *Mallotus* (data set 2). Area nomenclature follows Fig. 1.

The number of optimised areas per internal node only occasionally showed differences for 2, 3 or 4 areas per node. This occurred for nodes for which the optimisation was already very ambiguous (many possibilities, all with a low probability, shown in black in Figs. 6 and 7). The historical biogeographical analyses show a different picture for each genus (Figs. 6 and 7; Tables 1 & 2). In general, the extant *Macaranga* species have a more limited distribution than the *Mallotus* species, which makes the optimisation for internal nodes less ambiguous for *Macaranga*. Tables 1 and 2 show the age (and interval) with the most likely ancestral areas per node for *Macaranga* and *Mallotus*, respectively. For both chronograms Borneo is resolved as the most likely ancestral area of the most recent common ancestor of *Macaranga* and *Mallotus* (area I in Fig. 6 – node 195 - and Fig. 7 - node 114). For *Mallotus* Borneo is the inferred ancestral area as well (Fig. 7 – node 113). *Macaranga* (Fig. 6 – node 164) has IMO as best optimisation, however, many different ones are present here, and most contain area I (Borneo).

*Macaranga* diversified on Borneo (nodes 108–111, Fig. 6), whereby *Macaranga lowii* became widespread in western Malesia and southeast Asia and the genus dispersed to Australia and New Caledonia (nodes 162, 163) between 32.72 (HPD 48.96–31.14) Ma (node 164 in Fig. 6) and 22.93 (HPD 35.02–11.92) Ma (Node 153 in Fig. 6). The clade starting with node 159 (nodes mainly optimised for Sumatra, area G, but most contemporary species occurring in other or more widespread areas, Fig. 6) became widespread in west Malesia, and a lineage dispersed eastward and radiated in the Moluccas/New Guinea area (area L, clade starting with node 127, Fig. 6). In the latter clade *Macaranga tanarius* dispersed back to western Malesia and southeastern Asia and the ancestral lineage leading to *Macaranga grandifolia* and *M. angustifolia* is inferred to have spread to the Philippines and Sulawesi (areas J and K, node 114). The clade with crown node 159 (Fig. 6), which dispersed to southeast Asia (node 158), dispersed from there further to Africa (area A) and Madagascar (area B). Within the African clade *Macaranga indica* dispersed back to southeast Asia.

The recovered reconstruction for *Mallotus* is more complex to interpret. From node 113 (Fig. 7) one clade (starting with node 112) developed mainly in east Asia (area D). This clade is characterised by pioneer species and a number of them is widespread, in some cases reaching Australia and New Caledonia. The second branch at node 113 splits into an early dispersal to New Guinea and Australia (areas L and M, nodes 65–67, Fig. 7) and a mainly Asian-west Malesian clade (starting with crown node 101). Within the latter, besides some widespread species, dispersal to east Malesia and Australia occurred twice in the small clade *Mallotus connatus-M. trinervius* (nodes 71–73, Fig. 7) and in the clade *Mallotus macularis-M. claoxyloides* (nodes 88–86). This group also contains *Mallotus subulatus* and *Mallotus oppositifolius*, which are inferred to be independent dispersals to Africa and Madagascar (areas A, B; Fig. 7).

## Discussion

### General

The sample sizes (57 species of *Macaranga* in data set 1 and 50 species of *Mallotus* in data set 2) are relatively small, including ca. 20% of the *Macaranga* species and 37% of the *Mallotus* species. Therefore, the results still have a high level of uncertainty and should be interpreted with caution, e.g., many of the recently evolved myrmecophytic *Macaranga* species are lacking [16], [17], which might mean that the lineage through time plot (Fig. 4) could show additional increases in recent speciation rates. Much has been done to create data sets that could be tested against each other. The data sets were independent with regards to the DNA sequences used and only partly overlap in sampling and calibration points. Therefore, it was unexpected to find a considerable difference between the crown node age estimates of *Mallotus* in data set 2 (34 Ma; Fig. 3) and data set 1 (56 Ma, Fig. 2). Perhaps this is also the reason for the somewhat deviating LTT of *Mallotus* (Fig. 5). One most likely reason is that the taxon sampling in data set 1 (Fig. 2) is far less complete and the phylogeny of *Mallotus* based on it differs considerably from that of the far more complete data set 2 (Fig. 3). Another explanation is that the genetic markers, different per set, may have quite different evolutionary rates. Also, the relationships between several *Mallotus* clades in Fig. 2 are quite different from those in Fig. 3 (the latter compares with the phylogeny published in [2]). Moreover, reconstructing the phylogeny of data set 1 with BEAST appeared to be difficult. Several extra monophyletic groups had to be defined, otherwise *Macaranga* ended up as part of the *Mallotus* clade instead as sister group (a result formerly obtained in a phylogeny reconstruction based on morphological data [45]). Because of congruence in phylogeny and biogeography between *Macaranga* (data set 1, Fig. 2) and *Mallotus* (data set 2, Fig. 3), see rest of discussion, data set 2 was selected to represent the *Mallotus* data, and those in data set 1 (Fig. 2) were ignored. Then the results of molecular divergence time estimates and ancestral area reconstructions of the two independent analyses corroborate each other and are in line with the geological record and palaeohistory of the distributional range of the study groups.

Because of the incomplete sampling, reconstructing the complete historical biogeography is not possible at this point. But even if all species were included, any analysis would still be based only on contemporary species distributions. From the fossil record we know that the modern day species distributions are incomplete as *Mallotus* was present on New Zealand in the Miocene ([23], see for a further interpretation [24]). However, Nucete et al. [24] show that none of the other fossil records outside the current generic distributions can be reliably identified as *Macaranga* and/or *Mallotus* (and these were not used as calibration points). This means that only distribution modelling of palaeontological distributions might give some idea about former distributions, but most of the climate data, especially for the early Neogene and the Paleogene, are very rough. Therefore, such reconstructions were not attempted at this time.

### Selection of analyses and calibration points

In both data sets the oldest calibration points were 86.4 Ma (HPD 90–81 Ma, nodes 197 and 117 in Fig. 2 and 3, respectively) based on [22]. In the BEAST analyses the age of the nodes are 87.83 (HPD 90.00–83.67) Ma for *Macaranga* (Fig. 2) and 83.67 (HPD 90.00–82.00) Ma for *Mallotus* (Fig. 3). The differences in age between both genera fall just within the HPD limits.

In the analysis of *Macaranga* and *Mallotus*, the calibration point b (‘New Zealand’) was used (31–15 Ma) in both data sets. The corresponding node for the *Macaranga* analysis is the crown node of *Mallotus discolor* and *Mallotus pleiogynus* (Fig. 2), which is estimated to be 24.52 (HPD 29.45–15.00) Ma. In the *Mallotus* analysis it is node 67 (crown node of *Mallotus nesophilus*, *M. discolor*, *M. chromocarpus* and *M. pleiogynus*; Fig. 3) with an age of ca. 22.62 (HPD 24.17–15.00) Ma.

The ‘Africa’ calibration point c, crown node 156 (Fig. 2), only used in the *Macaranga* analysis, was set at 32–22 Ma. The age estimate by BEAST for this node was ca. 22.48 (HPD 23.80–22.00) Ma, which just falls within the range of the calibration.

The third calibration point (d) in the *Mallotus* analysis was the ‘Japan’ fossil of 42 (49–27) Ma, placed at the crown node of *Mallotus philippensis* and *Mallotus repandus* (node 103 in Fig. 3). Here we find the largest deviation from the fossil age, BEAST estimated the age of this node at ca. 29.66 (HPD 34.13–27.00) Ma. Moving the calibration point to the stem node of all pioneer species, node 113 (Fig. 3), would only change the estimated age to 34.31 (HPD 44.79–32.35) Ma. This might have been a better position as Tanai [26], [27] also pointed at relationships between the ‘Japan’ fossil and the pioneer species. However, the latter could not be done, because the monophyly of all pioneer species is still disputable (e.g., polyphyletic in Fig. 2). There is a discrepancy in divergence times for *Mallotus* between Fig. 2 and Fig. 3 (see beginning of discussion), the times in Fig. 2 are older, but this is not the case for the *Mallotus philippinensis-M. repandus* node, nor for the pioneer species (*Mallotus paniculatus-M. tetracoccus*).

### Historical Biogeography

Both data sets seem to generate similar historical biogeographical scenarios, with an emphasis on Borneo-west Malesia-mainland southeast Asia and several dispersals to Australia/west Pacific, Japan and Africa. But the question is how likely these scenarios are, and whether they match with the geological record. Borneo is the most probable ancestral area for the crown node of the *Macaranga*+*Mallotus* clade [node 195 in Fig. 2, 63.82 (HPD 63.33–79.13) Ma, Paleocene; node 114 in Figs. 3 and 7, 53.32 (HPD 69.57–48.25) Ma, Early Eocene]. The *Macaranga* crown node (node 164 in Fig. 6) is 32.72 (HPD 48.96–31.14) Ma and has many possible optimisations, all with a low probability, of which the ones with the highest probabilities contain Borneo (area I, next to Australia, M, and New Caledonia, O). The crown node of *Mallotus* (node 113 in Fig. 7) is 34.31 (HPD 44.79–32.35) Ma and has Borneo as optimisation. At those times, (the south-western part of) Borneo formed Sundaland with Sumatra and the Malay Peninsula and Southeast Asia [46]–[48]. The Philippines and East Malesia (and Java) had not emerged.

Both genera dispersed from Borneo to Southeast Asia, or they first became widespread and then underwent vicariance. For *Macaranga* this happened in the period between 30–18 Ma, in Fig. 6 between node 161 [29.68 (HPD 41.46–28.01)] and 111 [17.61 (HPD 24.54–8.08) Ma] and for *Mallotus* in the period from 35–32 Ma, between node 113 [34.31 (HPD 44.79–32.35) Ma] and node 112 [32.13 (HPD 40.04–29.04) Ma] in Fig. 7. Speciation in *Mallotus* is somewhat older and appears more extensive than in *Macaranga* at the time of reaching Japan. The clade of *Mallotus* containing the pioneer species (crown node 112 in Fig. 7) was mostly widespread, with several lineages crossing Wallace's line and reaching New Guinea and Australia. The exact timing of these events is unknown, but may be relatively recent.

The two genera show an early clade dispersing to New Guinea, Australia and New Caledonia. In *Macaranga* (with extinction in New Guinea) this probably occurred somewhere between stem node 164 [32.72 (HPD 48.96–31.14) Ma] and crown node 163 [22.93 (HPD 35.02–11.92) Ma], and for *Mallotus* between stem node 102 [33.24 (HPD 42.43–30.11) Ma] and crown node 67 [22.62 (HPD 24.17–15.00) Ma]. Although the temporal concurrence is evident, it is not easy to link it to specific geological events. There is a lack of consensus as to whether various terranes were completely [48] or partially submerged and available to act as stepping stones [49]. Hall (pers. comm.) admits that for geologists it is difficult to indicate whether or not a microplate was (temporarily) above water. Hall [50] showed that the Australian plate (together with east Malesia and New Guinea) was nearing west Malesia and floral exchange was possible, but in his reconstructions of areas above water [48], it appeared that only chains of volcano arcs would provide a pathway to Australia (in Hall's reconstructions New Guinea was still under water except for some small areas). Van Ufford & Cloos [51] indicate that a large eustatic fall in sea level of about 90 m occurred during 33–30 Ma (Oligocene) and resulted in several areas emerging, e.g., the Siga Formation had periods of aerial exposure as plant fossils and coal films were found in its type locality, the Bird's Head. Vicariance and dispersals back and forth between Australia and New Caledonia occurred often [52].

The next major split in *Macaranga* is between a mainly New Guinean clade (area L), reached between stem node 160 [26.60 (HPD 34.79–25.28) Ma; Fig. 6) and crown node 127 [20.17 (HPD 28.69–13.78) Ma; Fig. 6), and a west Malesian clade, mainly optimised for Sumatra (area G), but with most species present on Borneo [crown node 159 (25.26 Ma, HPD 31.86–24.13 Ma); Fig. 6]. The New Guinean clade is a second major dispersal event to New Guinea within *Macaranga*. This clade shows a few widespread species; *Macaranga involucrata* is present from Sulawesi up to the west Pacific (areas KLMN), *Macaranga grandifolia* (Borneo, Sulawesi, areas JK) and *Macaranga hispida* (Philippines, Sulawesi, Moluccas-New Guinea, areas JKL) cross Wallace's line, while *Macaranga tanarius* dispersed even back to the Asian mainland (areas D to N). These appear to be individual dispersal events of contemporary species and may be relatively recent.

The situation in *Mallotus* is different (Fig. 7) with no distinct split into an Asian and New Guinean clade at node 101 [31.81 (HPD 40.51–28.22) Ma; Fig. 7], but both clades (crown nodes 84 and 100, Fig. 7) comprise 2 clades or 1 clade that dispersed to New Guinea-Australia, respectively. One clade, stem node 73 [20.77 (HPD 26.52–15.34) Ma] agrees with the dispersal age of the second *Macaranga* New Guinean clade. The crown node of the same *Mallotus* clade [node 72, 8.61(HPD 16.23–6.37) Ma; Fig. 7] agrees with the other two dispersal events in *Mallotus*: *Mallotus polyadenos* [node 69, 6.29 (HPD 14.84–4.86) Ma; Fig. 7] and the *Mallotus macularis-ficifolius* clade [stem node 89, 7.96 (HPD 16.77–4.65) Ma; Fig. 7]. These younger ages also agree with most estimated ages for the nodes of the *Macaranga* New Guinean clade (nodes 112–126 in Table 1, mainly indicating ‘local speciation’). At 20 Ma parts of East Malesia already had moved in such places that stepping stones between West Malesia and New Guinea appeared to be in place (see reconstructions in [50]), only large parts were probably still not above water [48]. Still, dispersal to New Guinea was possible and obviously occurred (perhaps via the outer Melanesian Arc [53]). New Guinea itself has a very complex history of area accretions [54], which seemingly offered opportunities for both genera to speciate in New Guinea. Van Ufford & Cloos [51] and Baldwin et al. [54] indicate that Peninsular orogeny started in the Oligocene (35–30 Ma) as a result of a collision with the Inner Melanesian Arc and the orogeny of the central mountain range began in the latest middle Miocene, at least 12 Ma, a collision with the Outer Melanesian Arc. Both agree with the speciation and dispersal in the youngest phylogenetic parts of *Macaranga* and *Mallotus*. A close comparison between speciation and area ontogeny is not made as many New Guinean species, especially in *Macaranga*, are lacking. The upper clade of *Mallotus* (crown node 84, Fig. 7) also contains a few widespread species, two of these, *Mallotus peltatus* and *M. resinosus* dispersed independently from west Malesia to New Guinea. *Mallotus tiliifolius*, like *Macaranga tanarius*, probably dispersed back to west Malesia.

### Africa

The lower *Mallotus* clade of crown node 100 (Fig. 7) contains two dispersals to Africa and Madagascar (areas A and B, Fig. 1). These seem only to entail individual species, *Mallotus subulatus* and *Mallotus oppositifolius*. Both are in the same clade (starting with node 100 in Fig. 7) and the species below their nodes of origin have mainly Southeast Asia as optimisation, though several are also present in south and east Asia (area D). This makes it likely that dispersal occurred from south(east) Asia to Africa and Madagascar. Both *Mallotus* species probably dispersed independently, but it may have occurred during the same period. Unfortunately, because it concerns individual species, the period is rather imprecise. *Mallotus oppositifolius* may have dispersed between the age of node 96 [28.54 (HPD 36.08–24.47) Ma; Fig. 7] and present. *Mallotus subulatus* may have dispersed in the period of node 91 [18.30 (HPD 28.59–15.80 Ma); Fig. 7] and node 90 [14.93 (HPD 18.71–5.29) Ma].

In *Macaranga*, node 157 (Fig. 6) is the crown node of the African-Madagascan species [23.39 (25.46–22.05) Ma]. This makes it likely that the dispersal occurred synchronous in *Macaranga* and *Mallotus*, somewhere at the end of the Oligocene (node 91, Fig. 7 for *Mallotus subulatus*, node 157, Fig. 6, for *Macaranga*, and somewhat indiscriminate for *Mallotus oppositifolius*, after 28.54 Ma). In both genera the local outgroups to the African species are all from SE Asia main land (areas D and E, Fig. 1).

The dispersal direction, Asia to Africa, is contrary to the rafting theory of India [7], [8], which brought taxa from Africa to Asia. The boreotropical forests hypothesis [9]–[11] covers the periods Paleocene and Eocene, which are older than when the *Macaranga* and *Mallotus* dispersals most likely took place. Previous identification of fossils attributed to *Macaranga* and/or *Mallotus* found in present day northern temperate regions could also not be confirmed [24]. The presence of a fossil *Macaranga* [25] in the Horn of Africa, which resembles the extant *Macaranga kilimandscharica* [24], is consistent with the existence of a dispersal route for *Macaranga* from Asia via southwest Asia and the Arabian peninsula [15], [55] in the early to middle Miocene when the climate was warm and humid [56]. The connectivity between Africa and Asia was good [55], [57], especially because land bridges between Africa and Southwest Asia occurred in the same time span (Meswa Bridge ca. 23.5 Ma and the *Gomphoterium* Bridge > 18 Ma [55]). Complete land bridges are not necessary for dispersal in both genera, stepping stones (areas not too far from each other) are enough, just as with the dispersal from West Malesia to New Guinea and Australia.

The crown group of African *Macaranga* [23.39 (HPD 25.46–22.05) Ma] splits into a continental African clade (nodes 149–156 in Fig. 6) and a Madagascan clade (nodes 143–148 in Fig. 6). Geologically and geographically the most logical dispersal (and speciation) occurred from Asia to continental Africa and then to Madagascar, partly because of the fossil in the Horn of Africa and partly because of Samonds et al. [58]. Samonds et al. indicated that Madagascar received mammal lineages predominantly from Africa up until 15–20 Ma, thus the time period that *Macaranga* and *Mallotus* dispersed from Asia to Africa and Madagascar. After 15–20 Ma the prevailing sea currents shifted and favoured immigration from Asia. It may well be that ancestral distributions were widespread, entailing both Africa and Madagascar, which was followed by vicariance in *Macaranga*. This would explain the presence of two *Macaranga* sister clades, one in Africa and one in Madagascar. In the two *Mallotus* species the vicariance never occurred and both are still widespread.

It is remarkable that the Madagascan *Macaranga* clade contains a species that dispersed back to Asia (*Macaranga indica*), which is sister to a species occurring on both Madagascar (area B in Fig. 1) and the Mascarene islands (area C in Fig. 1), *Macaranga mauritiana*. The presence on the islands makes dispersal back via continental Africa, Arabia and Southwest Asia rather unlikely. Probably, *Macaranga indica* (or its ancestor) reached India via long distance dispersal across the Indian Ocean [12], [13], for instance via the various island arcs [1], [14].

In the above, generally no real distinction is made between vicariance and dispersal. One reason is that S-DIVA commonly assumes a wide distribution range for ancestral species via dispersal followed by vicariance between the descending species. Moreover, it is impossible with our data to distinguish between a widespread distribution divided by vicariance or dispersal to another area with speciation at the same time. The former assumes a widespread ancestor, while the latter assumes dispersal (and thus speciation) in a descending species (occurring one node higher in the area cladogram). Here, dispersal is often assumed based on geological knowledge: merging (micro)plates/terranes, microplates emerging above water, orogenesis, etc., which precludes several widespread distributions (e.g., west Malesia – New Guinea before 25 Ma). Seemingly, *Macaranga* and *Mallotus* disperse well across water barriers as several contemporary species are very widespread (e.g., continental Asia to New Guinea), especially in *Mallotus*. This kind of long distance dispersal (across water barriers) is probably caused by birds, which likely resulted in a gradual extension of distributions. The fruits of both genera are typical for Euphorbiaceae, lobed, generally 3-locular capsules with a single seed per locule. The fruits are explosive once very dry and they may shed the seeds over a short distance. The pericarp is smooth or covered by short, soft spines. The fruit wall is thin and leathery and seeds lack any fleshy layer except for a few species of *Mallotus*, where a very thin aril may be present. Seed dispersal is seemingly never studied, no references were found, but Google and You Tube shows various pictures and movies of seed eating birds (www.besgroup.org/2011/12/26/feeding-behaviour-of-sunbirds/; www.besgroup.org/2009/11/06/macaranga-triloba-and-sunbirds/; www.youtube.com/watch?v=xIS2f5Suwdk). The birds likely act as dispersal agents. The reward for the birds may be the disc-like glandular hairs with which the fruits are covered and which act as extrafloral nectaries. Once dispersed, *Macaranga* mainly reacted with speciation, while *Mallotus* species became widespread. This is also more or less shown by the LTT plots. *Mallotus* (Fig. 5) starts speciation earlier than *Macaranga* (Fig. 4). Both show the highest speciation rates at about 20 Ma after which the curves more or less level off, more so in *Mallotus*. The latter agrees with the tendency of *Mallotus* for widespread species. However, the value of the LTT plots is limited due to the low sampling and they have not been use to draw conclusions. Especially recently evolved species are lacking, e.g., the myrmecophytic group of *Macaranga* [16], [17].

### Synchronicity between Macaranga and Mallotus

*Macaranga* and *Mallotus* are morphologically (see introduction) and phylogenetically (sister taxa) closely related. Therefore, we hypothesised that both genera will show considerable congruence in evolutionary development. If this is the case, then the dating and phylogeny reconstructions will have a higher credibility. Table 3 shows an overview, based on the discussion, of major dispersal events in *Macaranga* and *Mallotus*. It appears that in the majority of cases, both genera dispersed to the same areas at about the same time. There are differences though, with *Mallotus* apparently dispersing more easily than *Macaranga*, e.g., reaching isolated areas such as Japan and New Zealand, and with a higher proportion of species being widespread. On the other hand, *Macaranga* seemingly adapts more easily to local circumstances via speciation. This is also shown by recent molecular analyses of *Macaranga* species living in symbiosis with ants (myrmecophily), which show that genetic distances are minimal but species being distinct nevertheless [16], [17].

**Table 3:** Simultaneous dispersal events in the genera *Macaranga* and *Mallotus*.

Occurrence	*Macaranga*	*Mallotus*
Distribution crown node	Borneo (among others)	Borneo
Time	Oligocene	Oligocene
Dispersal Borneo to SE Asia	Oligocene – Miocene till recent	Oligocene till recent
Dispersal Borneo to New Guinea	Oligocene	Oligocene
Dispersal Borneo to New Guinea	Late Oligocene – Early Miocene	Early Miocene
New Guinea	Various speciations in Late Miocene	2 dispersals in Late Miocene
Asia to Africa and Madagascar	Early Miocene	Imprecise, can be Early Miocene
Widespread (Asia to New Guinea)	Mainly Pliocene and younger	Since Oligocene

## Conclusions

*Macaranga* and *Mallotus* show a high degree of temporal and geographical synchronicity in dispersal events. To some extent, this is to be expected, as the genera share very similar ecological strategies, have similar geographical distributions and a recent common ancestry. These may all lead to exposure and diversification under comparable biotic and abiotic conditions. In our study design, we paid particular attention to assemble DNA sequence data sets for *Macaranga* and *Mallotus* that were highly dissimilar. Confidence in biogeographical reconstruction and inferred, concordant dispersals increases when large-scale congruence exists in molecular dating results between the two data sets. Furthermore, we find that inferred dispersal events closely match known geological configurations and previously described dispersal pathways. Our study shows that concordant evolution with closely related species rich groups of Euphorbiaceae can progress rapidly, over large distances and in widely differing environments.

## Supporting Information

Appendix S1 Taxa used in BEAST analysis with three calibrated groups and the outgroup (O  =  Outgroup; A  =  African *Macaranga* clade; N  =  New Zealand *Mallotus* clade; J  =  Japan *Mallotus* clade; 1  =  Set 1  =  *Macaranga* and *Mallotus*; 2  =  Set 2  =  *Mallotus*). Areas (Fig. 1): A  =  Tropical Africa; B  =  Madagascar; C  =  Mascarene Islands; D  =  Pakistan-India (not Andaman/Nicobar Isl.) to S. China and Japan; E  =  Thailand (not Peninsular part), Laos, Cambodia, Vietnam; F  =  Peninsular Thailand, Malay Peninsula, Andaman and Nicobar Islands; G  =  Sumatra; H  =  Java; I  =  Borneo; J  =  Philippines; K  =  Sulawesi; L  =  Moluccas, New Guinea; M  =  Australia; N  =  West Pacific island chains; O  =  New Caledonia.
(DOCX)
