# Supplementary material for: Dated Phylogenies of the Sister Genera Macaranga and Mallotus (Euphorbiaceae): Congruence in Historical Biogeographic Patterns?
Source: PLoS One. 2014 Jan 17;9(1):e85713. doi: 10.1371/journal.pone.0085713 (PMC3894986; doi:10.1371/journal.pone.0085713)
Supplement: Appendix S1 — Taxa used in BEAST analysis with three calibrated groups and the outgroup (O = Outgroup; A = African Macaranga clade; N = New Zealand Mallotus clade; J = Japan Mallotus clade; 1 = Set 1 = Macaranga and Mallotus; 2 = Set 2 = Mallotus). Areas (Fig. 1): A = Tropical Africa; B = Madagascar; C = Mascarene Islands; D = Pakistan-India (not Andaman/Nicobar Isl.) to S. China and Japan; E = Thailand (not Peninsular part), Laos, Cambodia, Vietnam; F = Peninsular Thailand, Malay Peninsula, Andaman and Nicobar Islands; G = Sumatra; H = Java; I = Borneo; J = Philippines; K = Sulawesi; L = Moluccas, New Guinea; M = Australia; N = West Pacific island chains; O = New Caledonia. (DOCX) [file pone.0085713.s001.docx]

Taxa used in BEAST analysis with three calibrated groups and the outgroup (O = Outgroup; A = African *Macaranga* clade; N = New Zealand *Mallotus* clade; J = Japan *Mallotus* clade; 1 = Set 1 = *Macaranga* and *Mallotus*; 2 = Set 2 = *Mallotus*). Areas (Fig. 1): A = Tropical Africa; B = Madagascar; C = Mascarene Islands; D = Pakistan-India (not Andaman/Nicobar Isl.) to S. China and Japan; E = Thailand (not Peninsular part), Laos, Cambodia, Vietnam; F = Peninsular Thailand, Malay Peninsula, Andaman and Nicobar Islands; G = Sumatra; H = Java; I = Borneo; J = Philippines; K = Sulawesi; L = Moluccas, New Guinea; M = Australia; N = West Pacific island chains; O = New Caledonia.

| **Data Set** | **Taxa** | **Distribution** | **Outgroup** | **Africa** | **New Zealand** | **Japan** |
| --- | --- | --- | --- | --- | --- | --- |
| 1 | *Blumeodendron calophyllum* Airy Shaw | GIJ | O1 |  |  |  |
| 1&2 | *Blumeodendron kurzii* (Hook.f.) J.J.Sm. | FGHIJ | O1&2 |  |  |  |
| 1 | *Hancea acuminata* (Baill.) S.E.C.Sierra, Kulju & Welzen | B | O1 |  |  |  |
| 1 | *Hancea capuronii* (Léandri) S.E.C.Sierra, Kulju & Welzen | B | O1 |  |  |  |
| 1&2 | *Hancea eucausta* (Airy Shaw) S.E.C.Sierra, Kulju & Welzen | I | O1&2 |  |  |  |
| 1 | *Hancea griffithiana* (Müll.Arg.) S.E.C.Sierra, Kulju & Welzen | FI | O1 |  |  |  |
| 1 | *Hancea integrifolia* (Willd.) S.E.C.Sierra, Kulju & Welzen | C | O1 |  |  |  |
| 1 | *Hancea penangensis* (Müll.Arg.) S.E.C.Sierra, Kulju & Welzen | FGIJKL | O1 |  |  |  |
| 1 | *Hancea? spinulosa* (McPherson) S.E.C.Sierra, Kulju & Welzen | B | O1 |  |  |  |
| 1&2 | *Hancea subpeltata* (Blume) M.Aparicio | EFGHI | O1&2 |  |  |  |
| 1 | *Macaranga albescens* L.M.Perry | L |  |  |  |  |
| 1 | *Macaranga alchorneoides* Pax & Lingelsh. | O |  |  |  |  |
| 1 | *Macaranga aleuritoides* F.Muell. | LN |  |  |  |  |
| 1 | *Macaranga alnifolia* Baker | B |  |  |  |  |
| 1 | *Macaranga angustifolia* Lauterb. & K.Schum. | L |  |  |  |  |
| 1 | *Macaranga auriculata* (Merr.) Airy Shaw | DEFIJ |  |  |  |  |
| 1 | *Macaranga barteri* Müll.Arg. | A |  | A1 |  |  |
| 1 | *Macaranga bicolor* Müll.Arg. | J |  |  |  |  |
| 1 | *Macaranga bifoveata* J.J.Sm. | L |  |  |  |  |
| 1 | *Macaranga* cf*. brachytricha* Airy Shaw | L |  |  |  |  |
| 1 | *Macaranga clavata* Warb. | LN |  |  |  |  |
| 1 | *Macaranga conifera* (Rchb.f. & Zoll.) Müll.Arg. | FGIJK |  |  |  |  |
| 1 | *Macaranga densiflora* Warb. | LN |  |  |  |  |
| 1 | *Macaranga denticulata* (Blume) Müll.Arg. | DEFGH |  |  |  |  |
| 1 | *Macaranga diepenhorstii* (Miq.) Müll.Arg. | FG |  |  |  |  |
| 1 | *Macaranga domatiosa* Airy Shaw | L |  |  |  |  |
| 1 | *Macaranga ducis* Whitmore | L |  |  |  |  |
| 1 | *Macaranga echinocarpa* Baker | B |  |  |  |  |
| 1 | *Macaranga gabunica* Prain | A |  | A1 |  |  |
| 1&2 | *Macaranga gigantea* (Rchb.f. & Zoll.) Müll.Arg. | FGI |  |  |  |  |
| 1 | *Macaranga grallata* McPherson | B |  |  |  |  |
| 1 | *Macaranga grandifolia* (Blanco) Merr. | JK |  |  |  |  |
| 1 | *Macaranga heterophylla* (Müll.Arg.) Müll.Arg. | A |  | A1 |  |  |
| 1 | *Macaranga heynei* I.M.Johnst. | FG |  |  |  |  |
| 1 | *Macaranga* cf*. hispida* (Blume) Müll.Arg. | JKL |  |  |  |  |
| 1 | *Macaranga hullettii* King ex Hook.f. | FGI |  |  |  |  |
| 1 | *Macaranga hurifolia* Beille | A |  | A1 |  |  |
| 2 | *Macaranga hypoleuca* (Rchb.f. & Zoll.) Müll.Arg. | FGI |  |  |  |  |
| 1&2 | *Macaranga inamoena* F.Muell. | M |  |  |  |  |
| 1 | *Macaranga indica* Wight | DEFG |  |  |  |  |
| 1 | *Macaranga induta* L.M.Perry | L |  |  |  |  |
| 1 | *Macaranga involucrata* (Roxb.) Baill. | KLMN |  |  |  |  |
| 1 | *Macaranga klaineana* Pierre | A |  | A1 |  |  |
| 2 | *Macaranga kurzii* (Kuntze) Pax & K.Hoffm. | DE |  |  |  |  |
| 1 | *Macaranga lamellata* Whitmore | I |  |  |  |  |
| 1 | *Macaranga lowii* King ex Hook.f. var. *kostermansii* Airy Shaw | I |  |  |  |  |
| 1 | *Macaranga lowii* King ex Hook.f. var. *lowii* | DEFGIJ |  |  |  |  |
| 1 | *Macaranga mauritiana* Bojer ex Müll.Arg. | C |  |  |  |  |
| 1 | *Macaranga monandra* Müll.Arg. | A |  | A1 |  |  |
| 1 | *Macaranga novoguineensis* J.J.Sm. | L |  |  |  |  |
| 1 | *Macaranga oblongifolia* Baill. | B |  |  |  |  |
| 1 | *Macaranga obovata* Boivin ex Baill. | B |  |  |  |  |
| 1 | *Macaranga pachyphylla* Müll.Arg. | FGHI |  |  |  |  |
| 1 | *Macaranga pearsonii* Merr. | I |  |  |  |  |
| 1 | *Macaranga poggei* Pax | A |  | A1 |  |  |
| 1 | *Macaranga praestans* Airy Shaw | I |  |  |  |  |
| 1 | *Macaranga puncticulata* Gage | FGI |  |  |  |  |
| 1 | *Macaranga quadriglandulosa* Warb. | LN |  |  |  |  |
| 1 | *Macaranga rhizinoides* (Blume) Müll.Arg. | GH |  |  |  |  |
| 1 | *Macaranga saccifera* Pax | A |  | A1 |  |  |
| 1 | *Macaranga schweinfurthii* Pax | A |  | A1 |  |  |
| 1 | *Macaranga siamensis* S.J.Davies | E |  |  |  |  |
| 1 | *Macaranga strigosissima* Airy Shaw | I |  |  |  |  |
| 1 | *Macaranga subdentata* Benth. | M |  |  |  |  |
| 1&2 | *Macaranga tanarius* (L.) Müll.Arg. | DEFGHIJKLMN |  |  |  |  |
| 1 | *Macaranga tessellata* Gage | L |  |  |  |  |
| 1&2 | *Macaranga trichocarpa* (Zoll.) Müll.Arg. | EFGI |  |  |  |  |
| 1 | *Macaranga triloba* (Thunb.) Müll.Arg | FGHJ |  |  |  |  |
| 1 | *Macaranga umbrosa* S.J.Davies | I |  |  |  |  |
| 1 | *Macaranga winkleri* Pax & K.Hoffm. | I |  |  |  |  |
| 2 | *Mallotus apelta* (Lour.) Müll.Arg. | DE |  |  |  |  |
| 1&2 | *Mallotus barbatus* Müll.Arg. | DEFGH |  |  |  |  |
| 1&2 | *Mallotus brachythyrsus* Merr. | I |  |  |  |  |
| 2 | *Mallotus calocarpus* Airy Shaw | E |  |  |  |  |
| 1&2 | *Mallotus caudatus* Merr. | I |  |  |  |  |
| 2 | *Mallotus chromocarpus* Airy Shaw | L |  |  | N2 |  |
| 1&2 | *Mallotus claoxyloides* (F.Muell.) Müll.Arg. | LM |  |  |  |  |
| 1&2 | *Mallotus connatus* M.Aparicio | IJ |  |  |  |  |
| 2 | *Mallotus coudercii* (Gagnep.) Airy Shaw | E |  |  |  |  |
| 1&2 | *Mallotus cumingii* Müll.Arg. | IJK |  |  |  |  |
| 1&2 | *Mallotus decipiens* Müll.Arg. | DEF |  |  |  |  |
| 1&2 | *Mallotus discolor* F.Muell. ex Benth. | M |  |  | N1&2 |  |
| 2 | *Mallotus dispersus* P.I.Forst. | M |  |  |  |  |
| 1&2 | *Mallotus ficifolius* (Baill.) Pax & K.Hoffm. | M |  |  |  |  |
| 1&2 | *Mallotus glomerulatus* Welzen | E |  |  |  |  |
| 2 | *Mallotus japonicus* (Thunb.) Müll.Arg. | D |  |  |  |  |
| 1&2 | *Mallotus khasianus* Hook.f. | DE |  |  |  |  |
| 2 | *Mallotus korthalsii* (Scheff.) Müll.Arg. | FGHIJ |  |  |  |  |
| 1&2 | *Mallotus lackeyi* Elmer | IJ |  |  |  |  |
| 2 | *Mallotus lanceolatus* (Gagnep.) Airy Shaw | EF |  |  |  |  |
| 1&2 | *Mallotus leucocalyx* Müll.Arg. | EFJK |  |  |  |  |
| 2 | *Mallotus longinervis* M.Aparicio | I |  |  |  |  |
| 1&2 | *Mallotus macrostachyus* (Miq.) Müll.Arg. | EFGI |  |  |  |  |
| 2 | *Mallotus macularis* Airy Shaw | L |  |  |  |  |
| 2 | *Mallotus megadontus* P.I.Forst. | M |  |  |  |  |
| 2 | *Mallotus metcalfianus* Croizat | DE |  |  |  |  |
| 2 | *Mallotus minimifructus* S.E.C.Sierra | IJK |  |  |  |  |
| 1&2 | *Mallotus miquelianus* (Scheff.) Boerl. | FGIJ |  |  |  |  |
| 2 | *Mallotus mollissimus* (Geiseler) Airy Shaw | GHIJKLMN |  |  |  |  |
| 2 | *Mallotus montanus* (Müll.Arg.) Airy Shaw | F |  |  |  |  |
| 1&2 | *Mallotus muticus* (Müll.Arg.) Airy Shaw | FGI |  |  |  |  |
| 2 | *Mallutus nepalensis* Müll.Arg. | D |  |  |  |  |
| 2 | *Mallotus nesophilus* Müll.Arg. | M |  |  | N2 |  |
| 1&2 | *Mallotus nudiflorus* (L.) Kulju & Welzen | DEFGHIJ |  |  |  |  |
| 1&2 | *Mallotus oppositifolius* (Geiseler) Müll.Arg. | AB |  |  |  |  |
| 1&2 | *Mallotus pallidus* (Airy Shaw) Airy Shaw | E |  |  |  |  |
| 1&2 | *Mallotus paniculatus* (Lam.) Müll.Arg. | DEFGHIJKLM |  |  |  |  |
| 1&2 | *Mallotus peltatus* (Geiseler) Müll.Arg. | DEFGHIJKL |  |  |  |  |
| 1&2 | *Mallotus philippensis* (Lam.) Müll.Arg. | DEFGHIJKLMN |  |  | J2 |  |
| 1&2 | *Mallotus pierrei* (Gagnep.) Airy Shaw | E |  |  |  |  |
| 1&2 | *Mallotus pleiogynus* Pax & K.Hoffm. | L |  |  | N1&2 |  |
| 1&2 | *Mallotus polyadenos* F.Muell. | LM |  |  |  |  |
| 1&2 | *Mallotus repandus* (Rottler) Müll.Arg. | DEFGHIJKLO |  |  | J2 |  |
| 1&2 | *Mallotus resinosus* (Blanco) Merr. | DEHIJKLM |  |  |  |  |
| 1&2 | *Mallotus rhamnifolius* (Willd.) Müll.Arg. | D |  |  |  |  |
| 2 | *Mallotus sphaerocarpus* (Miq.) Müll.Arg. | G |  |  |  |  |
| 1&2 | *Mallotus subulatus* Müll.Arg. | A |  |  |  |  |
| 1&2 | *Mallotus tetracoccus* (Roxb.) Kurz | DE |  |  |  |  |
| 1 | *Mallotus thorelii* Gagnep. | E |  |  |  |  |
| 2 | *Mallotus tiliifolius* (Blume) Müll.Arg. | DFGHIJKLMN |  |  |  |  |
| 2 | *Mallotus trinervius* (Lauterb. & K.Schum.) Pax | L |  |  |  |  |
